# Short sleep is associated with higher prevalence and increased predicted risk of cardiovascular diseases in an Iranian population: Fasa PERSIAN Cohort Study

**DOI:** 10.1038/s41598-020-61506-0

**Published:** 2020-03-12

**Authors:** Mohammad Hosein Yazdanpanah, Reza Homayounfar, Ali Khademi, Fariba Zarei, Ali Shahidi, Mojtaba Farjam

**Affiliations:** 1 Student Research Committee, Fasa University of Medical Sciences, Fasa, Iran; 2 Noncommunicable Diseases Research Center, Fasa University of Medical Sciences, Fasa, Iran

**Keywords:** Health care, Medical research, Risk factors, Cardiovascular diseases

## Abstract

Cardiovascular disease (CVD) is the leading cause of death worldwide. One common factor that may affect CVD risk factors is sleep disturbance. The factors influencing an individual’s sleep may vary among different cultures. The current study investigated sleep quality and quantity in the Fasa cohort population as an Iranian population. In a cross-sectional study using the Fasa PERSIAN cohort study data, 10,129 subjects aged 35–70 were entered. Self-reported sleep duration and cardiovascular events were recorded. The Framingham risk score (FRS) is used to predict cardiovascular events. Adjusted logistic regression showed significant odds ratios in subjects who sleep less than 6 hours for CVD (OR = 1.23; 95% CI:1.03–1.48), coronary heart disease (CHD) (OR = 1.21; 95% CI:1.009–1.46), and hypertension (HTN) (OR = 1.37; 95% CI:1.16–1.62). Higher risk profiles were also seen in the FRS for short sleepers. The highest significant odds ratios in FRS profiles in the intermediate high-risk group compared with the low-risk group were (1.44; 95% CI:1.18–1.75) in CVD and (1.48; 95% CI:1.16–1.88) in CHD risk score profiles. It can be suggested that participants with short durations of sleep had significantly higher CVD, HTN prevalence, and 10-year FRS. Participants with long sleep durations had no increase in CVD, CHD, myocardial infarction (MI), or HTN prevalence. MI prevalence was at the lowest level in subjects who got 8 to 8.9 hours of sleep.

## Introduction

Cardiovascular disease (CVD) comprises the most common disorders in both developed and developing countries. CVDs result in disability and reduced efficiency and can also have an economic burden on regional health systems^1^. For example, more than $231 billion US were spent on personal healthcare due to CVD in 2013^2^. Recent studies have shown that the prevalence of CVD is increasing in Central and Eastern Europe^3^. In 2015, cardiovascular disease caused more than 176,000 deaths in Iran^4^. There is no accurate data on CVD mortalities (including myocardial infarction (MI) and coronary heart disease (CHD)) in Iran; however changes in the lifestyle of Iranian people are related to a progressive increase in the prevalence of CVDs in Iran^5^.

The World Health Organization (WHO) recently recommended a global strategy to control and prevent non-communicable diseases (like CVDs) based on the reduction of exposure to risk factors^6^. Age, gender, blood pressure, lipid profiles, physical activity, obesity, smoking, and type 2 diabetes are some of the common risk factors for CVD.

Factors that may affect CVD directly or its risk factors are sleep disturbances^7,8^. Sleep deprivation can be associated with obesity^9^, diabetes^10^, and hypertension (HTN)^11,12^. This association can be a predisposing factor for the incidence of cardiac disease^13^.

According to the U.S. National Sleep Foundation, 31% of Americans in 2001 sleep 6 hours or less daily^14^. People today sleep 1.5 fewer hours than people who lived in the past century. Evidence shows that inadequate sleep has massive effects on both physiological and mental health^15^. Research has shown that sleep deprivation and, to a lesser extent, oversleeping result in the incidence of chronic diseases, while sleeping between 7 and 8 hours per night has the least effect on it^16^.

The Framingham risk score (FRS) is a popular scoring system for the calculation of the future risk of developing CVD in the next 10 years^17^. It was first developed based on the data resulted from a cohort study performed on the population residing in the city of Framingham, Massachusetts. Only one study carried out in Korea and based on the Korean National Health and Nutrition Examination Survey (KNHANES) on individuals older than 18 years of age reported that both short sleep (less than 5 hours) and long sleep (more than 8 hours) affected an individual’s FRS and gave the study population a moderate to severe probability for developing CVD in the next 10 years^18^.

According to another study carried out on Japanese people, short sleep duration (less than 7 hours) is associated with CVD^19^. According to a 2012 study on an Australian population older than 45 years of age, oversleeping causes a rise in the occurrence of CVD, especially among older people^20^. A group of studies in the U.S. showed a direct correlation between sleep deprivation and oversleeping and developing CVD, while another group of studies in the U.S. could not prove any correlation between sleep and cardiac diseases^21^.

The factors influencing an individual’s sleep duration may vary between different cultures and countries^22,23^, and these variations may change the correlation between sleep characteristics and health risks. For example, in the past decade, the sleep duration of adults in the U.S. decreased more compared to adults in Finland^24,25^. Similar studies have yet to be carried out in Iranian communities, and it would be of special interest to see if the previous findings could be reproduced in a large Iranian population. Because several studies have reported controversial results, there is still a knowledge gap due to uncertainty and lack of investigation of the relationship between sleep duration and specific types of CVD (CHD, MI), HTN, and FRSs (CHD, MI, CVD, CHD death, and CVD death risk scores).Thus, the current study investigated the relationship between sleep duration and various CVD-affected participants and different risk scores including those based on FRS in a population-based cohort study (Fasa PERSIAN Cohort Study) in order to reduce the problems and expenses related to CVD by comprehending the effects of sleep on these diseases.

## Results

### Demographics

This study evaluated 10,129 subjects, and their baseline characteristics are reported in Table 1. The mean sleep duration was 6.82 ± 0.66 hours in men and 6.97 ± 1.59 hours in women, which is a significant difference (*p*-value < 0.0001). The mean of age of the total population was 48.63 ± 9.57 years. The maximum mean age among sleep duration groups was seen in females who sleep less than 6 hours. The lowest BMI and the highest MET means were seen in males who sleep more than 9 hours. Overall, BMI and age with a negative correlation coefficient and HDL and DBP with a positive one had a significant relation with sleep duration with *p*-values of <0.0001 and 0.007, respectively. All variable correlations with sleep duration are reported in Appendix A.

**Table 1:** Baseline characteristics of subjects based on gender and sleep duration.

Variables	Male						Female						Total					
	Sleep Duration (Hours)						Sleep Duration (Hours)						Sleep Duration (Hours)					
	–5.9	6.0–6.9	7.0–7.9	8.0–8.9	9.0–	P-value	–5.9	6.0–6.9	7.0–7.9	8.0–8.9	9.0–	P-value	–5.9	6.0–6.9	7.0–7.9	8.0–8.9	9.0–	P-value
N (%)	969 (21.2)	985 (21.5)	1218 (26.6)	880 (19.2)	523 (11.4)	—	1011 (18.2)	1164 (20.9)	1487 (26.8)	1180 (21.2)	712 (12.8)	—	1980 (19.5)	21.49 (21.2)	2705 (26.7)	2060 (20.3)	1235 (12.2)	—
Age (Years)	49.10 ± 9.35	48.49 ± 9.23	48.89 ± 9.76	48.04 ± 9.63	48.23 ± 10.38	0.103^a^	51.76 ± 9.19	48.99 ± 9.18	47.63 ± 9.39	47.44 ± 9.64	47.76 ± 9.75	**<0.001**^**a**^	50.46 ± 9.36	48.76 ± 9.20	48.20 ± 9.58	47.69 ± 9.64	47.96 ± 10.02	**<0.001**^a^
Smokers (%)	412 (42.5)	357 (36.2)	450 (36.9)	370 (42.0)	222 (42.4)	**0.004**^**b**^	31 (3.1)	28 (2.4)	26 (1.7)	26 (2.2)	21 (2.9)	0.217^b^	443 (22.4)	385 (17.9)	476 (17.6)	396 (19.2)	243 (19.7)	**0.001**^b^
Alcohol Drinkers (%)	51 (5.3)	34 (3.5)	59 (4.8)	33 (3.8)	32 (6.1)	0.080^b^	—	—	—	—	—	—	51 (2.6)	34 (1.6)	59 (2.2)	33 (1.6)	32 (2.6)	0.059^b^
Has Diabetes (%)	76 (7.8)	93 (9.4)	95 (7.8)	54 (6.1)	37 (7.1)	0.112^b^	190 (18.5)	173 (14.9)	242 (16.3)	167 (14.2)	119 (16.7)	**0.036**^**b**^	266 (13.4)	266 (12.4)	337 (12.5)	221 (10.7)	156 (12.6)	0.122^b^
BMI (Kg/m^2^)	24.56 ± 4.46	24.61 ± 4.53	24.15 ± 4.38	23.79 ± 4.26	23.15 ± 5.1	**<0.001**^**a**^	27.07 ± 4.88	26.92 ± 4.77	27.13 ± 4.84	26.61 ± 4.72	26.29 ± 4.81	**0.001**^**a**^	25.84 ± 4.84	25.86 ± 4.81	25.78 ± 4.87	25.41 ± 4.74	25.11 ± 4.77	**<0.001**^a^
MET	41.47 ± 11.54	41.97 ± 11.43	42.81 ± 12.40	43.13 ± 12.68	43.87 ± 13.44	**0.001**^**a**^	41.22 ± 11.03	40.71 ± 10.17	40.52 ± 10.61	40.19 ± 10.31	40.05 ± 10.71	0.119^a^	41.34 ± 11.28	41.29 ± 10.78	41.55 ± 11.50	41.44 ± 11.47	41.67 ± 12.09	0.866^a^
Systolic BP (mm Hg)	109.49 ± 17.77	110.40 ± 17.48	110.07 ± 18.00	110.96 ± 18.66	111.48 ± 19.05	0.235^a^	112.80 ± 20.43	111.63 ± 17.75	112.08 ± 19.41	111.89 ± 20.45	111.41 ± 19.51	0.586^a^	111.18 ± 19.24	111.07 ± 17.64	111.17 ± 18.81	111.49 ± 19.71	111.44 ± 19.31	0.948^a^
Diastolic BP (mm Hg)	73.68 ± 12.17	74.36 ± 11.69	73.75 ± 12.12	74.90 ± 12.77	74.92 ± 12.27	0.082^a^	74.81 ± 12.47	74.37 ± 11.37	75.19 ± 12.38	74.77 ± 12.98	74.99 ± 12.22	0.559^a^	74.26 ± 12.33	74.37 ± 11.52	74.54 ± 12.28	74.83 ± 12.89	74.96 ± 12.24	0.404^a^
Total Cholesterol (mg/dL)	176.34 ± 42.48	177.51 ± 45.75	173.26 ± 42.68	178.87 ± 39.70	179.28 ± 40.16	**0.013**^**a**^	186.95 ± 43.26	188.05 ± 42.48	190.64 ± 43.55	186.88 ± 42.25	188.35 ± 48.28	0.164^a^	181.76 ± 43.20	183.22 ± 44.31	182.81 ± 44.01	183.46 ± 41.36	184.50 ± 45.23	0.490^a^
HDL (mg/dL)	44.63 ± 12.74	44.46 ± 13.70	46.93 ± 15.58	48.53 ± 16.26	50.77 ± 18.02	**<0.001**^a^	50.84 ± 13.76	52.32 ± 15.51	54.27 ± 17.02	55.49 ± 19.70	54.80 ± 19.20	**<0.001**^a^	47.80 ± 13.63	48.72 ± 15.21	50.97 ± 16.79	52.52 ± 18.63	53.10 ± 18.80	**<0.001**^a^

Data are reported as mean ± standard deviation or as number (percentages). P-value reported as the result of ^a^Analysis of variance (ANOVA) and ^b^Chi-square. Statistically significant P-values are bolded(P-value < 0.05).

BMI = body mass index, BP = blood pressure, HDL = high density lipoprotein.

### Prevalence of CVD

Table 2 shows the prevalence of cardiovascular diseases and hypertension in the different sleep groups. Overall, 1,182 subjects reported a history of CVD, of whom 440 (37.23%) were men and 742 (62.77%) were women. The highest prevalence of CVD in the sleep duration groups was seen among those of both genders who sleep less than 6 hours. Higher prevalence rates for CVD, CHD, and HTN were seen in females who were short sleepers. MI also showed a greater prevalence rate in short sleepers, although it was in males. In the total study population, the prevalence rates of CVD, MI, CHD, and HTN among the sleep duration groups were significant with *p*-values of 0.005, 0.04, 0.008, and <0.0001, respectively. After gender distinction, higher prevalence rates of CVD, CHD, and HTN were seen in women.

**Table 2:** Prevalence of cardiovascular diseases and hypertension in different sleep groups and the association between these diseases and sleep duration according to gender.

	Male					female				total			
Prevalence of	Sleep duration (hours)	Number of cases (%)	p-value	OR (95% CI)	AOR* (95% CI)	Number of cases (%)	p-value	OR (95% CI)	AOR* (95% CI)	Number of cases (%)	p-value	OR (95% CI)	AOR* (95% CI)
**CVD**	–5.9	109/969 (11.2)	0.318	1.25 (0.94–1.65)	1.27 (0.96–1.69)	167/1011 (15.5)	**0.009**	**1.37 (1.09–1.72)**^**a**^	1.19 (0.94–1.51)	276/1980 (13.9)	**0.005**	**1.30 (1.09–1.55)**^**a**^	**1.23 (1.03–1.48)**^c^
	6.0–6.9	94/985 (9.5)		1.04 (0.78–1.39)	1.04 (0.78–1.40)	163/1164 (13.2)		1.13 (0.90–1.41)	1.11 (0.88–1.40)	257/2149 (12.0)		1.09 (0.91–1.30)	1.09 (0.91–1.31)
	7.0–7.9	112/1218 (9.2)		1.00 (reference)	1.00 (reference)	187/1487 (11.9)		1.00 (reference)	1.00 (reference)	299/2705 (11.1)		1.00 (reference)	1.00 (reference)
	8.0–8.9	74/880 (8.4)		0.90 (0.66–1.23)	0.89 (0.65–1.32)	142/1180 (11.2)		0.95 (0.75–1.20)	0.94 (0.74–1.19)	216/2060 (10.5)		0.94 (0.78–1.13)	0.94 (0.77–1.13)
	9.0–	51/523 (9.8)		1.06 (0.75–1.51)	1.04 (0.73–1.49)	83/712 (10.7)		0.91 (0.69–1.20)	0.89 (0.67–1.19)	134/1235 (10.9)		0.97 (0.78–1.21)	0.97 (0.77–1.21)
**MI**	–5.9	32/969 (3.3)	0.118	1.45 (0.86–2.42)	1.46 (0.87–2.45)	14/1011 (1.4)	0.435	0.98 (0.49–1.93)	0.76 (0.37–1.54)	46/1980 (2.3)	**0.040**	1.28 (0.85–1.93)	1.16 (0.77–1.76)
	6.0–6.9	19/985 (1.9)		0.83 (0.46–1.50)	0.82 (0.45–1.48)	16/1164 (1.4)		0.97 (0.50–1.87)	0.89 (0.45–1.75)	35/2149 (1.6)		0.89 (0.57–1.39)	0.86 (0.55–1.35)
	7.0–7.9	28/1218 (2.3)		1.00 (reference)	1.00 (reference)	21/1487 (1.4)		1.00 (reference)	1.00 (reference)	49/2705 (1.8)		1.00 (reference)	1.00 (reference)
	8.0–8.9	14/880 (1.6)		0.68 (0.36–1.31)	0.70 (0.36–1.34)	8/1180 (0.7)		0.47 (0.21–1.08)	0.46 (0.20–1.05)	22/2060 (1.1)		**0.58 (0.35–0.97)**^**a**^	**0.58 (0.35–0.97)**^c^
	9.0–	10/523 (1.9)		0.82 (0.39–1.71)	0.85 (0.40–1.77)	9/712 (1.3)		0.89 (0.40–1.96)	0.84 (0.38–1.87)	19/1235 (1.5)		0.84 (0.49–1.44)	0.83 (0.48–1.42)
**CHD**	–5.9	99/969 (10.2)	0.290	1.21 (0.91–1.62)	1.23 (0.92–1.65)	157/1011 (15.5)	**0.009**	**1.36 (1.07–1.71)**^**a**^	1.18 (0.93–1.50)	256/1980 (12.9)	**0.008**	**1.28 (1.07–1.53)**^**a**^	1.21 (1.009–1.46)^c^
	6.0–6.9	85/985 (8.6)		1.01 (0.75–1.36)	1.01 (0.75–1.38)	154/1164 (13.2)		1.12 (0.89–1.42)	1.10 (0.87–1.40)	239/2149 (11.1)		1.07 (0.89–1.29)	1.08 (0.89–1.30)
	7.0–7.9	104/1218 (8.5)		1.00 (reference)	1.00 (reference)	177/1487 (11.9)		1.00 (reference)	1.00 (reference)	281/2705 (10.4)		1.00 (reference)	1.00 (reference)
	8.0–8.9	65/880 (7.4)		0.85 (0.61–1.18)	0.84 (0.60–1.17)	132/1180 (11.2)		0.93 (0.73–1.18)	0.92 (0.72–1.18)	197/2060 (9.6)		0.91 (0.75–1.10)	0.91 (0.74–1.10)
	9.0–	49/523 (9.4)		1.10 (0.77–1.58)	1.09 (0.75–1.57)	76/712 (10.7)		0.88 (0.66–1.17)	0.86 (0.64–1.15)	125/1235 (10.1)		0.97 (0.77–1.21)	0.96 (0.76–1.21)
**HTN**	–5.9	125/969 (12.9)	0.390	1.14 (0.88–1.47)	1.24 (0.93–1.67)	350/1011 (34.6)	**<0.001**	**1.58 (1.33–1.89)**^**a**^	**1.44 (1.17–1.78)**^**a**^	475/1980 (24.0)	**<0.001**	**1.35 (1.17–1.55)**^**a**^	**1.37 (1.16–1.62)**^a^
	6.0–6.9	109/985 (11.1)		0.95 (0.73–1.25)	0.93 (0.69–1.27)	333/1164 (28.6)		**1.20 (1.01–1.42)**^**a**^	**1.30 (1.06–1.60)**^**b**^	442/2149 (20.6)		1.10 (0.96–1.27)	1.17 (0.99–1.38)
	7.0–7.9	140/1218 (11.5)		1.00 (reference)	1.00 (reference)	372/1487 (25.0)		1.00 (reference)	1.00 (reference)	512/2705 (18.9)		1.00 (reference)	1.00 (reference)
	8.0–8.9	89/880 (10.1)		0.86 (0.65–1.14)	0.81 (0.58–1.12)	286/1180 (24.2)		0.95 (0.80–1.14)	0.96 (0.78–1.19)	375/2060 (18.2)		0.95 (0.82–1.10)	0.94 (0.79–1.12)
	9.0–	55/523 (10.5)		0.90 (0.65–1.25)	0.82 (0.56–1.21)	166/712 (23.3)		0.91 (0.73–1.12)	0.88 (0.69–1.13)	221/1235 (17.9)		0.93 (0.78–1.11)	0.90 (0.73–1.11)

CVD = Cardiovascular diseases, MI = Myocardial infarction, CHD = Coronary heart disease, HTN = Hypertension. P-value reported as the results of chi-square test and Statistically significant P-values are bolded.

Frequency of each disease reported as number (percent).OR = odds ratio, CI = confidence interval, AOR = adjusted odds ratio, *adjusted for first three Principal Component Analysis.

Statistically significant ORs are bolded.

^a^Significant level at <0.001.

^b^Significant level at <0.01.

^c^Significant level at <0.05.

**Figure 1:**
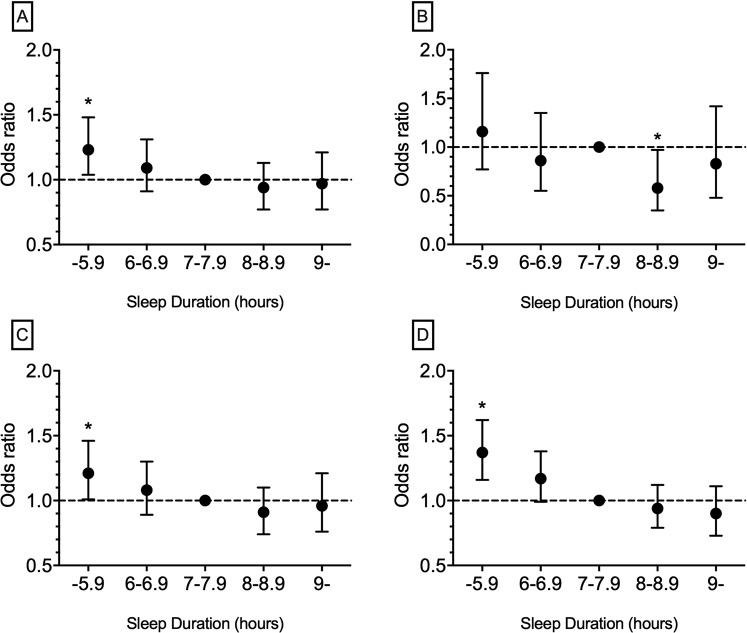
Odds ratios of prevalence in (**A**) cardiovascular diseases, (**B**) myocardial infarction, (**C**) coronary heart disease, and (**D**) hypertension according to sleep duration group among total population. *Statistically significant odds ratios (*p*-value < 0.05).

Furthermore, the odds ratios (ORs) related to CVD and HTN in different sleep duration groups are reported in Table 2. In MI, the highest OR was related to males who sleep less than 6 hours, and the lowest OR was related to women who sleep 8–8.9 hours compared to the reference group. For HTN and CHD, the highest ORs were seen in women who sleep less than 6 hours and the lowest ORs were seen in men who sleep 8–8.9 hours compared to the reference group. For CVD, the highest and lowest ORs belonged to women who sleep less than 6 hours and men who sleep 8–8.9 hours, respectively, compared to the reference group. Regardless of gender, significant ORs in HTN, CHD, and CVD (which had the highest OR among other groups) were related to the group who sleep less than 6 hours (*p*-value < 0.0001). For MI, those who sleep 8–8.9 hours showed a significant OR which was the smallest (*p*-value < 0.0001). All ORs (highest OR in HTN, CHD and CVD and lowest OR in MI) remained significant after multivariable adjustment (*p*-value < 0.05) (Table 2). Figure 1 shows the ORs for CVD, MI, CHD, and HTN prevalence rates in sleep duration groups among the total population.

### 10-year Framingham risk scores

In 10-year FRSs, CHD, MI, CVD, CHD death, and CVD death risks had significant negative relationships with sleep hours. All the Pearson correlation coefficients and their *p*-values for 10-year FRSs and the sleep duration correlation are reported in Appendix B. The means and standard deviations of 10-year FRSs (CVD, CHD, MI, CHD death, CVD death) in different sleep groups and the post hoc Tukey analysis results are provided in Table 3. Overall, the FRS was higher in groups with a shorter duration of sleep. CHD and MI with *p*-values < 0.0001, CVD with a *p*-value = 0.002, and CHD death with a *p*-value = 0.19 showed significant differences among sleep groups. CVD death did not show a significant difference between groups (*p*-value = 0.146). Considering gender, the difference in 10-year FRSs among sleep groups was seen only in women. The group with the short sleep duration (−5.9 hours) showed a significant difference with at least one other sleep group in all risk scores except CVD death.

**Table 3:** The means and standard deviations of different 10-year Framingham risk scores and the comparison of these scores in different sleep duration groups according to gender.

10-year Risk of (%)	Sleep duration (hours)	Male	Female	Total
CVD	–5.9	6.69 ± 7.21	2.50 ± 4.08	4.84 ± 6.38
	6.0–6.9	6.29 ± 7.52	1.98 ± 3.47	4.20 ± 6.29*
	7.0–7.9	6.44 ± 7.20	1.86 ± 3.22*	4.10 ± 5.99*
	8.0–8.9	6.45 ± 6.92	1.90 ± 3.37*	4.07 ± 5.82*
	9.0–	6.33 ± 7.08	1.99 ± 3.64	4.01 ± 5.92*
MI	–5.9	2.61 ± 3.24	0.47 ± 1.09	1.67 ± 2.74
	6.0–6.9	2.42 ± 3.40	0.37 ± 0.95	1.43 ± 2.73
	7.0–7.9	2.37 ± 3.20	0.32 ± 0.85*	1.33 ± 2.53*
	8.0–8.9	2.38 ± 3.02	0.33 ± 0.87*	1.31 ± 2.41*
	9.0–	2.32 ± 3.09	0.36 ± 1.03	1.27 ± 2.45*
CHD	–5.9	5.08 ± 4.99	1.76 ± 2.74	3.62 ± 4.46
	6.0–6.9	4.80 ± 5.17	1.40 ± 2.43	3.15 ± 4.41*
	7.0–7.9	4.83 ± 4.91	1.25 ± 2.18*	3.00 ± 4.18*
	8.0–8.9	4.85 ± 4.65	1.25 ± 2.26*	2.97 ± 4.02*
	9.0–	4.66 ± 4.68	1.31 ± 2.46*	2.87 ± 4.03*
CHD Death	–5.9	0.70 ± 1.19	0.14 ± 0.51	0.45 ± 0.99
	6.0–6.9	0.68 ± 1.38	0.08 ± 0.30	0.39 ± 1.05
	7.0–7.9	0.67 ± 1.27	0.07 ± 0.32*	0.36 ± 0.96
	8.0–8.9	0.65 ± 1.18	0.07 ± 0.29*	0.35 ± 0.89*
	9.0–	0.63 ± 1.33	0.09 ± 0.41	0.34 ± 0.99
CVD Death	–5.9	0.84 ± 1.41	0.25 ± 0.77	0.58 ± 1.21
	6.0–6.9	0.82 ± 1.57	0.16 ± 0.46*	0.50 ± 1.22
	7.0–7.9	0.84 5 ± 1.51	0.14 ± 0.47*	0.48 ± 1.16
	8.0–8.9	0.84 7 ± 1.54	0.15 ± 0.49*	0.48 ± 1.17
	9.0–	0.849 ± 1.81	0.19 ± 0.76	0.49 ± 1.40

Data are reported as mean ± standard deviation. CVD = Cardiovascular diseases, MI = Myocardial infarction, CHD = Coronary heart disease.

*Significant difference with group −5.9 as results of post hoc Tukey analysis (P-value < 0.05).

Among the total population, 992 subjects with CVD, 158 subjects with MI, 588 subjects with CHD, 428 subjects with CHD death, and 120 subjects with CVD death were in the intermediate-high risk group. There was a significant difference in frequency between the groups with low and intermediate-high risk for CVD and CHD (*p*-value = 0.001). After gender distinction, the only significant difference between the low and intermediate-high risk groups was seen among men. Although the highest frequency in intermediate-high risk profiles in MI and CHD death was seen in short sleepers, it was not significant (*p*-value > 0.05).

**Figure 2:**
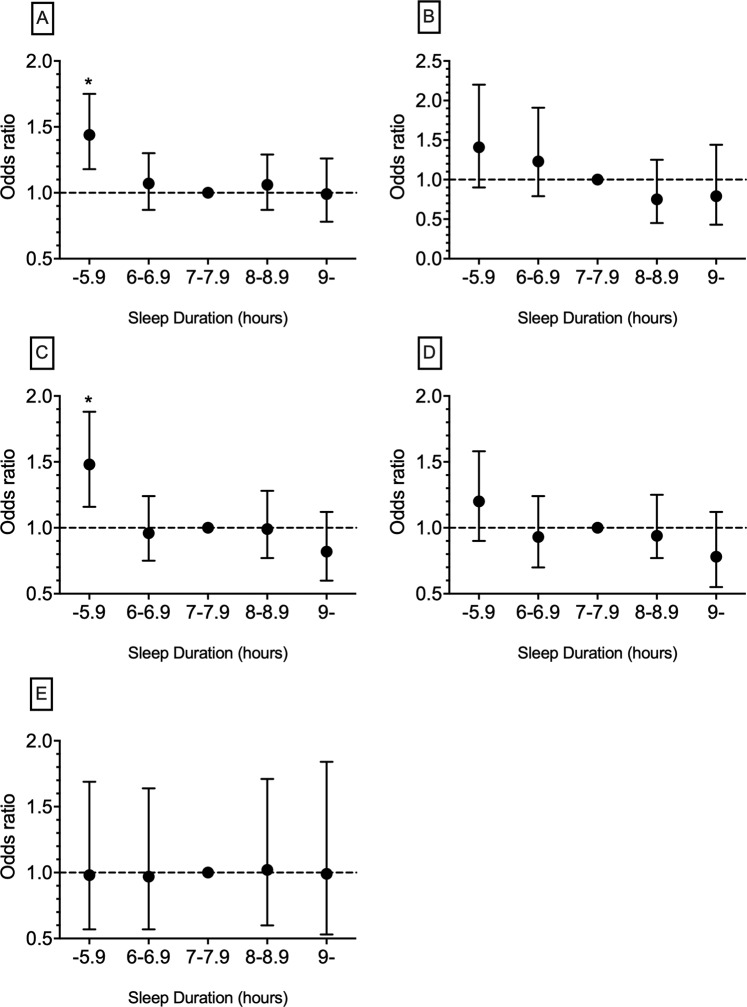
Odds ratios of intermediate-high-risk profiles compared to low-risk profiles of 10-year Framingham risk scores in (**A**) cardiovascular diseases, (**B**) myocardial infarction, (**C**) coronary heart disease, (**D**) coronary heart disease death, and (**E**) cardiovascular disease death according to sleep duration group in total population. *Statistically significant odds ratios (*p*-value < 0.05).

Table 4 presents the ORs for intermediate-high risk compared with low risk groups in different 10-year FRSs among sleep duration groups according to gender and the total population. In all 10-year FRSs except CVD death, the highest ORs were observed in the short sleep group (−5.9 hours), and only ORs in CVD and CHD were statistically significant (*p*-value < 0.0001). In the case of CVD death, no increase in OR was observed. Figure 2 shows the ORs of intermediate-high risk compared with low risk in 10-year FRSs according to sleep duration group in the total population.

**Table 4:** Predicted risk of cardiovascular diseases based on 10-year Framingham risk scores in different sleep groups and the association between these risk scores and sleep duration according to gender.

Framingham risks of	Sleep duration (hours)	Male			Female			Total		
		Low risk n (%)	Intermediate-high risk n (%)	OR (95% CI)	Low risk n (%)	Intermediate-high risk n (%)	OR (95% CI)	Low risk n (%)	Intermediate-high risk n (%)	OR (95% CI)
CVD	–5.9	589 (75.1)	192 (24.9)	1.20 (0.96–1.50)	572 (93.9)	37 (6.1)	**2.03 (1.25–3.30)**^**a**^	1372 (83.4)	229 (16.6)	**1.44 (1.18–1.75)**^**a**^
	6.0–6.9	644 (79.1)	170 (20.9)	0.96 (0.76–1.20)	733 (95.7)	33 (4.3)	1.41 (0.76–2.32)	1148 (87.1)	203 (12.9)	1.07 (0.87–1.30)
	7.0–7.9	782 (78.4)	215 (21.6)	1.00 (reference)	1008 (96.9)	32 (3.1)	1.00 (reference)	1787 (87.9)	247 (12.1)	1.00 (reference)
	8.0–8.9	585 (78.1)	164 (21.9)	1.02 (0.81–1.28)	787 (95.5)	37 (4.5)	1.48 (0.81–2.39)	1367 (87.2)	201 (12.8)	1.06 (0.87–1.29)
	9.0–	341 (78.8)	92 (21.2)	0.98 (0.74–1.29)	477 (96.0)	20 (4.0)	1.32 (0.74–2.33)	815 (87.9)	112 (12.1)	0.99 (0.78–1.26)
MI	–5.9	734 (95.1)	39 (4.9)	1.20 (0.76–1.89)	608 (99.8)	1 (0.2)	N.A.	1341 (97.2)	39 (2.8)	1.41 (0.90–2.20)
	6.0–6.9	776 (95.3)	39 (4.7)	1.14 (0.72–1.79)	765 (99.9)	1 (0.1)	N.A.	1541 (97.5)	39 (2.5)	1.23 (0.79–1.91)
	7.0–7.9	956 (95.9)	41 (4.1)	1.00 (reference)	1040 (100)	0	N.A.	1995 (98)	41 (2)	1.00 (reference)
	8.0–8.9	725 (96.8)	24 (3.2)	0.77 (0.46–1.28)	824 (100)	0	N.A.	1549 (98.5)	24 (1.5)	0.75 (0.45–1.25)
	9.0–	418 (96.5)	15 (3.5)	0.83 (0.45–1.52)	497 (100)	0	N.A.	915 (98.4)	15 (1.6)	0.79 (0.43–1.44)
CHD	–5.9	639 (82.8)	133 (17.2)	1.27 (0.98–1.65)	595 (97.7)	14 (2.3)	2.01 (0.92–4.38)	1228 (89.3)	147 (10.7)	**1.48 (1.16–1.88)**^**a**^
	6.0–6.9	709 (87.1)	105 (12.9)	0.90 (0.69–1.19)	757 (98.8)	9 (1.2)	1.01 (0.42–2.42)	1463 (92.8)	114 (7.2)	0.96 (0.75–1.24)
	7.0–7.9	857 (86.0)	140 (14.0)	1.00 (reference)	1028 (98.8)	12 (1.2)	1.00 (reference)	1882 (92.5)	152 (7.5)	1.00 (reference)
	8.0–8.9	643 (85.8)	106 (14.2)	1.009 (0.76–1.32)	813 (98.7)	11 (1.3)	1.15 (0.50–2.64)	1454 (92.6)	117 (7.4)	0.99 (0.77–1.28)
	9.0–	385 (88.9)	48 (11.1)	0.76 (0.53–1.08)	487 (98.0)	10 (2.0)	1.75 (0.75–4.10)	872 (93.8)	58 (6.2)	0.82 (0.60–1.12)
CHD death	–5.9	687 (89.0)	85 (11.0)	0.97 (0.72–1.31)	599 (98.4)	10 (1.6)	**2.87 (1.04–7.95)**^**a**^	1286 (93.1)	95 (6.9)	1.20 (0.90–1.58)
	6.0–6.9	732 (89.9)	82 (10.1)	0.88 (0.65–1.19)	762 (99.5)	4 (0.5)	0.90 (0.42–3.21)	1494 (94.6)	86 (5.4)	0.93 (0.70–1.24)
	7.0–7.9	885 (88.8)	112 (11.2)	1.00 (reference)	1034 (99.4)	6 (0.6)	1.00 (reference)	1919 (94.2)	118 (5.8)	1.00 (reference)
	8.0–8.9	668 (89.2)	81 (10.8)	0.95 (0.70–1.29)	819 (99.4)	5 (0.6)	1.05 (0.50–3.46)	1487 (94.5)	86 (5.5)	0.94 (0.77–1.25)
	9.0–	396 (91.5)	37 (8.5)	0.73 (0.50–1.09)	491 (98.8)	6 (1.2)	2.10 (0.67–6.5)	887 (95.4)	43 (4.6)	0.78 (0.55–1.12)
CVD death	–5.9	753 (97.5)	19 (2.5)	0.78 (0.44–1.40)	606 (99.5)	3 (0.5)	N.A.	1359 (98.4)	22 (1.6)	0.98 (0.57–1.69)
	6.0–6.9	789 (96.9)	25 (3.1)	0.98 (0.57–1.68)	766 (100.0)	0 (0.0)	N.A.	1555 (98.4)	25 (1.6)	0.97 (0.57–1.64)
	7.0–7.9	966 (96.9)	31 (3.1)	1.00 (reference)	1038 (99.8)	2 (0.2)	N.A.	2004 (98.4)	33 (1.6)	1.00 (reference)
	8.0–8.9	724 (96.7)	25 (3.3)	1.07 (0.63–1.83)	823 (99.9)	1 (0.1)	N.A.	1547 (98.3)	26 (1.7)	1.02 (0.60–1.71)
	9.0–	420 (97.0)	13 (3.0)	0.96 (0.50–1.86)	495 (99.6)	2 (0.4)	N.A.	915 (98.4)	15 (1.6)	0.99 (0.53–1.84)

CVD = Cardiovascular diseases, MI = Myocardial infarction, CHD = Coronary heart disease. Frequency of risk score groups reported as number (percent). OR = odds ratio, CI = confidence interval, N.A. = not applicable due to zero frequency. Statistically significant ORs are bolded (P-value < 0.001).

## Discussion

### Main findings

The current study achieved several important findings. First, by categorizing individuals into 5 ranges of sleep duration, it was determined that the frequency of CVD, MI, CHD, and HTN prevalence was significantly higher in short sleepers. Second, an inverse correlation was observed between sleep duration and 10-year CVD, CHD, MI, CVD death, and CHD death risk score and was significant in women and the total population, but not in men. Third, there was an inverse correlation between sleep duration, age, and BMI. Fourth, a positive association was observed between sleep duration and DBP and HDL.

The current study focused on the prevalence of different CVDs, including MI, CHD, and HTN, based on sleep duration; an attempt was made to determine if a correlation between FRS and sleep duration existed in an Iranian population.

### Other findings

The current study revealed that the maximum mean age in both genders was found in the short sleep group, which is in agreement with the results of Im *et al*.^18^, but in contrast with the findings of Amagai *et al*.^26^, who reported that the maximum mean age was seen in the long sleep duration group (>9). Data in the current study suggested an inverse correlation between BMI and sleep duration. This study obtained findings similar to those of different studies^27,28^ which reported a correlation between short sleep duration and increased BMI. In line with these results, 1,024 subjects with short sleep duration in the Wisconsin Sleep Cohort Study showed increased BMI^27^. Conversely, another study found that the maximum mean BMI belonged to females who sleep more than 9 hours^26^. In the case of lipid profiles and sleep duration, total cholesterol and sleep duration had a positive but not significant correlation; however, Toyama *et al*. studied male employees in Japan and reported a negative significant correlation between these two factors (r = −0.13)^29^. In the same study, HDL had a positive association (r = 0.03) with sleep, although it was not statistically significant. The current study also suggested a positive significant correlation between HDL and sleep duration. No relationship between SBP and sleep duration was observed in the current study; however, Javaheri *et al*. showed that children with poor sleep quality (in which, sleep duration was accounted as a factor of sleep quality) had higher systolic blood pressure (about 4 mm Hg)^30^. In the current data, DBP showed a positive and significant correlation with sleep duration. It seems that subjects with a higher sleep duration had a higher diastolic blood pressure. This result was in contrast with the CARDIA cohort study among young adults aged 33 to 45 years^31^. The differences in results may be explained by the different ranges of age in that study compared to the current one (35–75 yr). Interestingly, the current study found a positive correlation between MET and sleep duration in men and a negative one in women, both of which were statistically significant.

### The prevalence of CVD

Data in the current study showed that the prevalence of CVD was increased by nearly 30% in men who sleep less than 6 hours, but in people with long sleep duration, this increase was just about 19% in comparison with the reference group. The significance of the adjusted OR in the group who sleep less than 6 hours compared with the reference group suggests short sleep duration may be an independent risk factor for CVD prevalence. Sabanayagam *et al*. reported a greater increase in the prevalence of CVD among men with short sleep durations than in those with long sleep durations^32^. This result supports the results of the current study. However, in their study, this increase was more prominent among women, which does not agree with the current findings. Overall, no significant increase in the prevalence of CVD in both genders and in the total population was suggest for those with long sleep duration. In the case of MI prevalence, there was just a significant adjusted OR in the 8–8.9-hour sleep duration group compared with the reference group, which can suggest an independent protective factor in MI prevalence. One study reported that shorter sleep duration is associated with a greater prevalence of MI^33^, which supports the increasing prevalence of MI (16%) among short sleepers reported in the current study. In the same study, longer sleep duration was associated with a greater prevalence of CHD and angina, which contradicted the current findings which emphasized no association between long sleep duration and CHD prevalence. Because there was a significant adjusted OR in the group with less than 6 hours sleep duration compared with the reference group, it can be said that short sleep duration is related to the greater prevalence of CHD.

### The prevalence of HTN

A meta-analysis reported a U-shaped relationship between sleep duration and HTN in cross-sectional studies^34^, while the current data indicated that short sleep duration (<6 hours) had a higher prevalence of HTN, and no increase in HTN prevalence among those with long sleep duration was seen. After gender distinction, both genders with short sleep were at a higher risk for HTN prevalence; however, the increase was significant only in women, which may be due to their higher lipid profiles and BMI and lower physical activity levels compared to men. It may also be related only to the greater frequency of diabetes among women. Overall, the current study reported a significant 37% increase in HTN prevalence in short sleeper women, which cannot be ignored.

### The prediction of CVD using the framingham risk score

The current study showed that the prediction of intermediate and high CVD 10-year risk scores was significant and more than two times higher in females who sleep less than 6 hours compared to the reference group; however, this increase in men was only about 20%, which was not significant. There are several cohort studies about sleep duration and incidence of CVD and mortality with results indicating no relationship^35^ to results indicating a 3-times higher risk in sleep disturbances. The MONICA/KORA Augsburg Cohort Study included 6,896 adults and reported that in the group who got 5 or fewer hours of sleep, the risk of MI was 3 times higher, but only in females^36^. The Nurses’ Health Study of 71,617 women aged 40 to 65 years showed that short sleep duration is associated with increased CHD risk^14^. Although these two studies support the current data, some studies have reported a relation between short sleeping and cardiovascular events to be greater in men. Kripke *et al*. observed that sleeping 4 hours or less was associated with more deaths, including those from cardiovascular causes, surprisingly among men^37^. Amagai *et al*. reported the same thing in men, but in those who sleep less than 6 hours^26^. There are some studies in contrast with the current one, which have reported that there is no association between an increased risk of mortality in either gender and short sleep duration^38–40^.

For long sleepers, it was observed that the intermediate and high CVD 10-year risk scores were greater than 30% in females who sleep 9 hours or more compared to those who sleep 7–7.9 h. An increase in risk among men, however, was not observed. A study of 82,969 women aged 40 to 65 years reported a 1.6-times increase in mortality among women who sleep 9 or more hours compared with those who sleep 7 hours, which was supportive of data from the current study^41^. Some studies, however, have also reported this relationship in men. In a 10-yr. follow-up of NHNES I cohort consisting of 7844 men and women aged older than 31, a 1.5-times higher risk of stroke for those who sleep more than 8 hours was seen compared with those who sleep 6–8 hours^21^. Another study reported an increased risk of CVD death for elderly people who were long sleepers (10 hours or more) regardless of gender^40^.

In general, both short sleep duration (more than 2 times) and long sleep duration (more than 1.3 times) had higher frequencies in the intermediate-high 10-year CVD risk group in women compared with the reference group. These results are also supported by Chen *et al*., who reported both associations of both long and short sleep durations with the risk of stroke as a cardiovascular disease in 93,175 women^42^. Data from the current study suggested that short sleep duration in women and in the total population may be an independent factor for higher CVD risk.

A doubled increase in CHD among female subjects was observed for short sleepers, and a 75% risk increase was observed for long sleepers, but the data was not statistically significant. However, among 71,617 U.S. female health professionals aged 45–65 years, there was an increase in CHD risk for short and long sleep durations^14^. An explanation for why the current data was not significant may be the small number of subjects in the intermediate-high risk group in the 10-year CHD risk profile. A 27% increase among male subjects in the short sleep group was also observed, but it was not significant. However, if gender in the CHD risk profile is not considered, a significant OR is obtained which shows a near 50% increase in CHD risk among those sleeping less than 6 hours compared with the reference group.

One can strengthen the analysis by claiming that the FRS is a limited value in the Iranian population. However, Khalili *et al*. studied the validity of the FRS as a screening tool and emphasized that its score is of equal value among Iranian and non-Iranian populations^43^. Only one study focused on FRS and sleep duration^18^, however, the study did not report mean of FRS, it lacked the comparison of FRSs among sleep duration groups, and no gender distinction was made. Essentially, the results concerning the short sleep group in that study agreed with the current results. In that study, the ORs of intermediate to high risk was 1.34 with a 95% CI (1.20–1.50) for the short sleep group (5 hours or less) and 1.14 with a 95% CI (1.01–1.32) for the long sleep group (9 hours or more) compared to the 6–8-h group. For comparison, the current study had a higher OR in the short sleep group and a lower OR in the long sleep group. Both studies were in line with each other regarding the short sleep group but not for the long sleep group. The first study obtained an OR of 1.34 with a 95% CI (1.060–1.472) in the prevalence of CVD for men in the short sleep group, which was near the current results, but this agreement was not seen in the results regarding women.

### Probable mechanism

There is some evidence for the probably mechanism of sleep on CVD, including neuroendocrine, metabolic, and gene expression effects and even anatomical changes in the heart. Various studies have reported that short sleep duration leads to reduced leptin levels^27,44^ and increased ghrelin^27^. It can also impact the hypothalamus axis^45^ and sympathovagal balance^46^, and increase sympathetic nervous system activity and cortisol levels^45^. Nishihara *et al*. reported that lower sleep efficiency leads to higher epinephrine excretion^47^. Another possible mechanism is the elevation of catecholamine in cardiovascular disease^48^. In metabolic effects, studies have observed that short sleep duration results in impaired glucose tolerance and reduced insulin sensitivity^45^, and higher levels of hemoglobin A1c^49^, total cholesterol^28,50^, triglycerides^28,51^, and LDL^50^. It is also associated with high blood pressure which can lead to HTN^11,52^. Tochikubo *et al*. conducted a short-term experiment and reported a significant increase in blood pressure in the short sleep duration group (mean 3.6 hours) in comparison with the sufficient sleep group (mean 8 hours)^53^. Inflammatory markers, including C-reactive protein^54^, TNF-α, and IL-6^55^, can also be mediators in CVD risk and lead to atherosclerosis. In the case of gene expression, there are sleep-related transcripts related to cholesterol metabolism^56^. Similarly, other studies have proposed that the circadian system has a direct role in lipid metabolism^57,58^. Balasubramaniam *et al*. reported that hepatic LDL-receptor gene expression is related to circadian rhythms^59^. Guindalini *et al*. observed the upregulation of glycerol-3-phosphate dehydrogenase enzyme gene expression as a mediator enzyme in carbohydrate and lipid metabolism^60^. Anatomical changes in the heart can also occur, such as an increase in wall thickness of more than 1.2 mm^61^.

Although the mechanism is unclear, changes in lipid profiles and increases in inflammatory markers related to long sleep duration^51,62^ have been reported. Other factors such as depression and socioeconomic status should not be ignored. Long sleep may just be a consequence of other diseases or their subclinical states^63,64^.

### Sleep: an independent or age-dependent risk factor for CVD?

Modifiable risk factors for CVD in the Framingham study include HDL, total cholesterol, SBP, smoking, and diabetes which can be modified to decrease the risk. Alternatively for this risk, sleep can be included as a modifiable risk factor^65^, and sleep durations can be modified to decrease the 10-year CVD risk.

Perhaps it is just the effect of age in sleep and FRS. According to previous studies, the duration of sleep is decreased with increasing age. Total sleep duration was significantly shorter in elderly subjects (8.13 hours) compared with middle-aged (9.06 hours) and young (10.53 hours) subjects^66^. In calculating the FRS, the effect of age has been boldly accounted for; for example, a BP > 160 mmHg and total cholesterol >130 mg/dL in males have lower points than the age of 50 years and more. This can be interpreted to mean that the current findings are more attributable to increased age rather than decreased sleep duration.

### Limitations

The current work is not free of limitations. This is a cross-sectional study and cannot declare a causative element for any of the factors influencing others. To propose a cause-and-effect process, close observation must be made at follow-up intervals during the Fasa cohort study, which can add to the value of the work. The current study was based on self-report by the individuals, and as a consequence the data is subjective, but Sankai *et al*. have suggested that self-report sleep is valid compared to actigraphy^67^. Factors affecting the quality of sleep, like sleep apnea, sleep latency, and other quality factors, were not evaluated in the current study; rather, focus was placed solely on the quantity of sleep. There is no documented cause for short sleep duration among individuals in the data; it could be the consequence of psychological or physical problems or just being exhausted from overwork. It is suggested that these factors be implemented during the follow-ups. The current study examined a rural population; it is suggested that a similar study be carried out in an urban community to determine any possible differences.

## Conclusion

Both parts of the current study (prevalence of CVD and Framingham risk calculation) indicated that short sleep duration in the total population had significantly higher CVD and CHD prevalence rates and higher frequencies in the intermediate and high 10-year CVD and CHD risk profiles. A significantly higher prevalence of HTN was also seen in those with short sleep duration in the total population. No increase in CVD, CHD, MI, or HTN prevalence, nor increased frequency of intermediate and high 10-year Framingham risk profiles was seen in long sleep duration. The current data also suggested a protective independent factor for MI prevalence: 8 to 8.9 hours of sleep.

## Method

### Population

This cross-sectional study used data from the Fasa Cohort Study as a branch of the PERSIAN cohort, a longitudinal study for evaluating non-communicable diseases^68–70^. From a total of 41,000 individuals in a rural region of Sheshdeh, subjects aged 35 to 70 years (11,097 participants) were considered as the study population. Data collection was conducted from 2015 to 2016, and each participant signed an informed consent at the beginning of the study process. All participants could withdraw from the Fasa Cohort Study as they wished. All collected data was stored in a codified database. Without considering ethnicity, all participants were at the same socioeconomic level. After excluding 968 subjects (8.72%) due to lack of data on sleep duration and regular use of hypnotic sedative drugs, 10,129 subjects ultimately took part in the study.

### Measurements

Demographic data including age and gender were recorded in the questionnaire, which also collected data on each participant’s current status of smoking, alcohol intake, and medical history including diabetes, HTN, and CVDs such as CHD, MI, and stroke. Each subject followed the protocol mentioned in reference 68^68^. Participant weight and height were measured by trained personnel and recorded. Height was measured using a stadiometer with an accuracy of 0.1 cm, and weight was measured using a digital scale with an accuracy of 0.1 kilograms (Tanita BC-418, Tanita Corp, Japan). Body mass index (BMI) was calculated by weight (Kg) divided by square of height (m). Trained personnel measured and recorded diastolic and systolic blood pressure twice with a 15-minute interval. The mean blood pressure was then reported as systolic blood pressure and diastolic blood pressure in mmHg. Samples of 10 mm of venous blood under conditions of 12-hour fasting were taken for measuring serum lipid profiles. Samples were transported to the Noncommunicable Diseases Research Center (NCDRC) laboratory under standard conditions, and a Mindray BS380 autoanalyzer (Mindray Medical International, Shenzhen, China) was used for biochemical testing.

Physical activity was evaluated using a 20-item questionnaire that included questions on common activities in the Iranian rural population. The time of each activity was recorded in hours and minutes. The final physical activity score was calculated by multiplying the MET coefficient of activities by the period of that activity in hours.

### Group categorization based on sleep duration measurements

Data on the night sleeping of subjects was collected using two items of the Pittsburgh Sleep Quality questionnaire^71^: “When do you usually go to bed? __”, “When do you usually get up in the morning? __”. Using these answers, the subject’s sleep duration per night was calculated. Subjects were categorized into 5 groups: sleep duration lower than 6 hours (−5.9), 6 to 6.9 hours (6–6.9), 7 to 7.9 hours (7–7.9), 8 to 8.9 hours (8–8.9), 9 and more than 9 hours (9.0–). In this study the 5.9 and 9.0- groups were considered as short and long sleep durations, respectively. The 7–7.9 group was set as the reference group.

### Framingham risk score calculation

**Figure 3:**
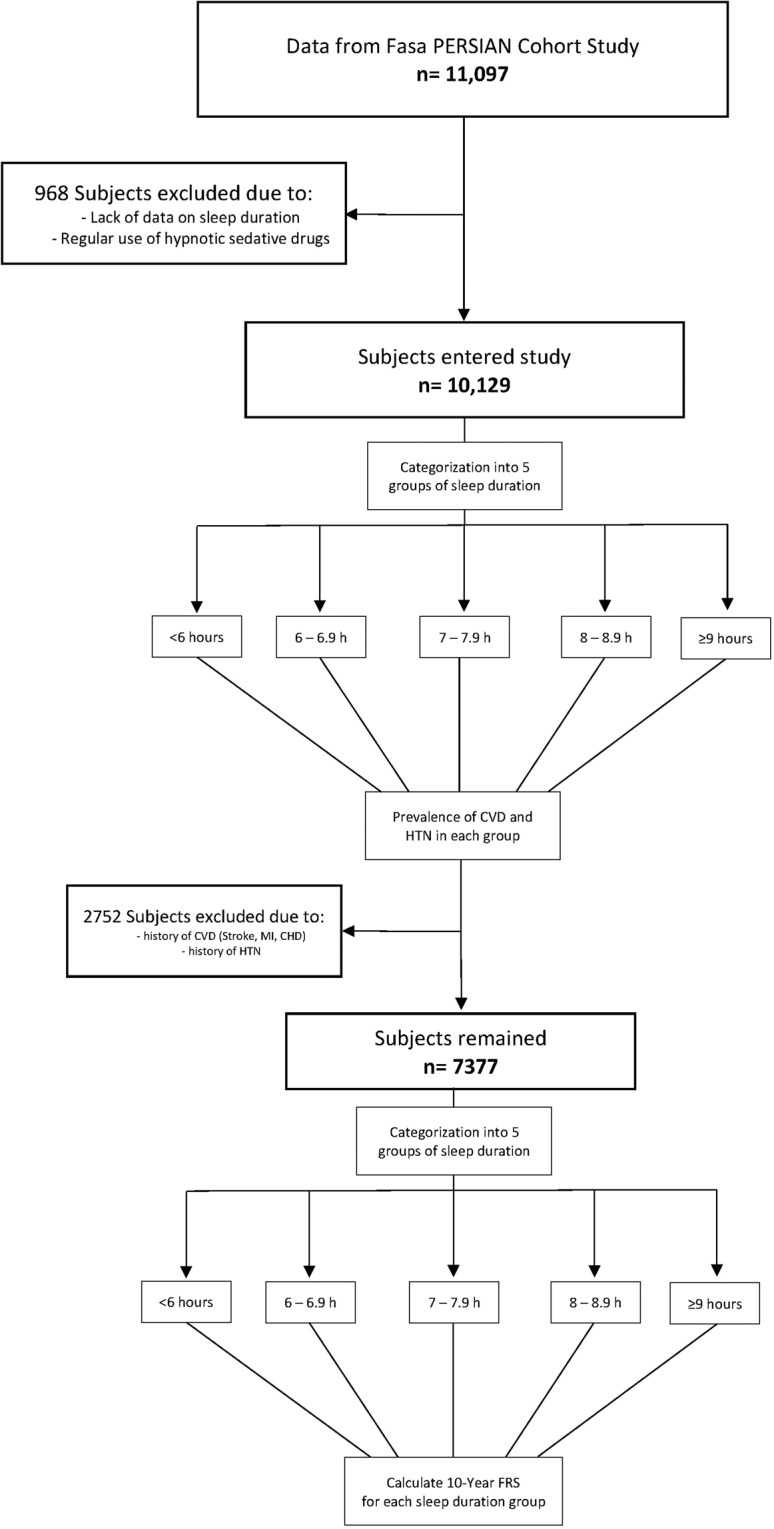
Flowchart of subject selection and study method.

The Framingham 10-year CVD risk formula was used for the calculation of CVD risk prediction. Before calculating, subjects with a history of CVD or HTN were excluded. Ultimately, 7377 subjects remained. Figure 3 shows an overview of the study method. The Framingham formula predicted a CVD risk in the following 10 years by cardiovascular risk factors including age, gender, HDL, cholesterol, systolic blood pressure, history of smoking, and history of diabetes. To compare low-risk profiles and intermediate- and high-risk profiles, subjects with less than 10% were considered low risk and with 10% or more were considered as intermediate-high risk and were grouped accordingly. The cut-off points for low and intermediate-high risk for CHD death and CVD death risk profiles were set 2% and 5%, respectively.

### Statistical analysis

All variables are reported as mean ± standard deviation, number (percentage). For comparison between two groups, the independent-samples t-test was used, and for the comparison of more than two groups, one-way ANOVA and post hoc Tukey analysis were used. For categorical variables, the chi-square test was performed. For the calculation of odds ratios (ORs) and a 95% confidence interval (CI), logistic regression was used. Nine variables including age, BMI, blood pressure, smoking, diabetes, lipid profiles, and physical activity levels were used to adjust the regression, and the intercorrelation between these variables affected the results of regression. To avoid collinearity, a principle component analysis (PCA) with varimax rotation was used. The first three components which had Eigenvalues greater than 1 covered 53% of the variance. The regression coefficients of the first three components were selected as the adjusting variables in multivariable logistic regression. The PCA results are reported in Appendix C. A significance level of *p*-value < 0.05 was considered, and all analyses were performed using IBM SPSS Statistics, version 23 (IBM Corp., Armonk, N.Y., USA). For forest plot graphs, Prism version 8.00 (GraphPad Software, La Jolla, California, USA) was used.

### Ethics approval and consent to participate

All experimental methods were performed according to the relevant guidelines and regulations of our regional and national research ethics committees. The protocol of this study was approved by the regional and national research ethics committees (the equivalent of institutional review boards) of FUMS, (reference number: IR.FUMS.REC.1397.167).

## Supplementary information

Supplementary information.
