# Supplementary material for: Short sleep is associated with higher prevalence and increased predicted risk of cardiovascular diseases in an Iranian population: Fasa PERSIAN Cohort Study
Source: Sci Rep. 2020 Mar 12;10:4608. doi: 10.1038/s41598-020-61506-0 (PMC7067883; doi:10.1038/s41598-020-61506-0)
Supplement: Supplementary file 1 — Supplementary information. [file 41598_2020_61506_MOESM1_ESM.docx]

**Appendix A**

| variable | Sleep Duration(hours) | | | | | |
| --- | --- | --- | --- | --- | --- | --- |
|  | male | | female | | total | |
|  | r | p | r | p | r | p |
| Age (years) | -.028 | (.056) | **-.150** | **(<0.001)** | **-.093** | **(<0.001)** |
| BMI (Kg/m^2^) | **-.076** | **(<0.001)** | **-.034** | **(.012)** | **-.037** | **(<0.001)** |
| SBP (mmHg) | **.046** | **(.002)** | -.019 | (.161) | .027 | (.242) |
| DBP (mmHg) | **.048** | **(.001)** | .007 | (.601) | **.027** | **(.007)** |
| TC (mg/dl) | .014 | (.346) | -.005 | (.696) | .012 | (.340) |
| HDL (mg/dl) | **.118** | **(<0.001)** | **.071** | **(<0.001)** | **.099** | **(<0.001)** |
| MeT | **.062** | **(<0.001)** | **-.031** | **(.021)** | .012 | (.241) |

**Table A. Correlation between variables and sleep duration according to sex**

BMI= body mass index, DBP= Diastolic blood pressure, SBP=Systolic blood pressure, TC= Total cholesterol, HDL= High density lipoprotein, MET= Metabolic equivalent

r= Pearson correlation, statistically significant Pearson correlations and P-values are bolded(P-value<0.05).

**Appendix B**

| 10-year Framingham  risk score of | Sleep duration(hours) | | | | | |
| --- | --- | --- | --- | --- | --- | --- |
|  | male | | female | | total | |
|  | r | p | r | p | r | p |
| CVD | -.014 | (.380) | **-.053** | **(.001)** | **-.046** | **(<0.001)** |
| MI | -.029 | (.074) | **-.046** | **(.005)** | **-.052** | **(<0.001)** |
| CHD | -.024 | (.143) | **-.067** | **(<0.001)** | **-.058** | **(<0.001)** |
| CHD death | -.019 | (.234) | **-.044** | **(.007)** | **-.039** | **(.001)** |
| CVd death | .002 | (.917) | **-.043** | **(.009)** | **-.026** | **(.026)** |

**Table B. Correlation between different 10-year Framingham risk scores and sleep duration according to sex.**

CVD= Cardiovascular diseases, MI= Myocardial infarction, CHD= Coronary heart disease.

r= Pearson correlation, statistically significant Pearson correlations and P-values are bolded(P-value<0.05).

**Appendix C**

| Component | Initial Eigenvalues | | |
| --- | --- | --- | --- |
|  | Total | % of Variance | Cumulative % |
| 1 | 2.282 | 25.354 | 25.354 |
| 2 | 1.303 | 14.476 | 39.830 |
| 3 | 1.187 | 13.191 | 53.021 |

**Table C1. The first three components and their Total Variance Explained**

|  | Component | | |
| --- | --- | --- | --- |
|  | 1 | 2 | 3 |
| BMI (kg/m^2^) | .216 | -.018 | .777 |
| Age (Years) | .573 | .097 | -.260 |
| DBP (mm Hg) | .849 | .089 | .155 |
| SBP (mm Hg) | .914 | .103 | .094 |
| Smoking status | -.056 | -.195 | -.692 |
| TC (mg/dL) | .075 | .787 | .133 |
| HDL (mg/dL) | .045 | .844 | -.045 |
| Diabetes | .290 | -.127 | .193 |
| MET | .043 | .017 | -.297 |

**Table C2. Rotated load factor of cardiovascular risk factor on the first three PCA component**

BMI= body mass index (kg/m^2^), DBP= Diastolic blood pressure (mmHg), SBP= Systolic blood pressure (mmHg), TC= Total cholesterol, HDL= High density lipoprotein (mg/dL), MET= Metabolic equivalent

|  | | MET | Diabetes | HDL | TC | Smoking | age | SBP | DBP | BMI |
| --- | --- | --- | --- | --- | --- | --- | --- | --- | --- | --- |
| PCA factor score 1 | Pearson Correlation | .043 | .290 | .045 | .075 | -.056 | .573 | .914 | .849 | .216 |
|  | P-value | **<0.001** | **<0.001** | **<0.001** | **<0.001** | **<0.001** | **<0.001** | **<0.001** | **<0.001** | **<0.001** |
| REGR factor score 2 | Pearson Correlation | .017 | -.127 | .844 | .787 | -.195 | .097 | .103 | .089 | -.018 |
|  | P-value | .093 | **<0.001** | **<0.001** | **<0.001** | **<0.001** | **<0.001** | **<0.001** | **<0.001** | .070 |
| REGR factor score 3 | Pearson Correlation | -.297 | .193 | -.045 | .133 | -.692 | -.260 | .094 | .155 | .777 |
|  | P-value | **<0.001** | **<0.001** | **<0.001** | **<0.001** | **<0.001** | **<0.001** | **<0.001** | **<0.001** | **<0.001** |

**Table C3. Correlation of each principle component with the cardiovascular risk factor**

BMI= body mass index (kg/m^2^), DBP= Diastolic blood pressure (mmHg), SBP=Systolic blood pressure (mmHg), HDL= High density lipoprotein (mg/dL), MET= Metabolic equivalent, TC= Total cholesterol
